# Integration of Power-Free and Self-Contained Microfluidic Chip with Fiber Optic Particle Plasmon Resonance Aptasensor for Rapid Detection of SARS-CoV-2 Nucleocapsid Protein

**DOI:** 10.3390/bios12100785

**Published:** 2022-09-23

**Authors:** Ting-Chou Chang, Aileen Y. Sun, Yu-Chung Huang, Chih-Hui Wang, Shau-Chun Wang, Lai-Kwan Chau

**Affiliations:** 1 Center for Nano Bio-Detection, National Chung Cheng University, Chiayi 621301, Taiwan; 2 Instant NanoBiosensors, Co., Ltd., Taipei 115010, Taiwan; 3 Department of Chemistry and Biochemistry, National Chung Cheng University, Chiayi 62102, Taiwan

**Keywords:** fiber optic biosensor, gold nanoparticle, localized surface plasmon resonance, aptamer, microfluidic chip, binding kinetics, SARS-CoV-2, nucleocapsid protein, COVID-19, point-of-care testing

## Abstract

The global pandemic of COVID-19 has created an unrivalled need for sensitive and rapid point-of-care testing (POCT) methods for the detection of infectious viruses. For the novel coronavirus SARS-CoV-2, the nucleocapsid protein (N-protein) is one of the most abundant structural proteins of the virus and it serves as a useful diagnostic marker for detection. Herein, we report a fiber optic particle plasmon resonance (FOPPR) biosensor which employed a single-stranded DNA (ssDNA) aptamer as the recognition element to detect the SARS-CoV-2 N-protein in 15 min with a limit of detection (LOD) of 2.8 nM, meeting the acceptable LOD of 10^6^ copies/mL set by the WHO target product profile. The sensor chip is a microfluidic chip based on the balance between the gravitational potential and the capillary force to control fluid loading, thus enabling the power-free auto-flowing function. It also has a risk-free self-contained design to avoid the risk of the virus leaking into the environment. These findings demonstrate the potential for designing a low-cost and robust POCT device towards rapid antigen detection for early screening of SARS-CoV-2 and its related mutants.

## 1. Introduction

With the increasing demand on point-of-care diagnostics, the integration of biosensors with microfluidics provides promising opportunities to fulfill the extra requirements in point-of-care testing (POCT) such as short analysis time, portability, low-cost device and chip, ease-of-use, and small amount of sample and reagents, in addition to the sensitivity and selectivity considerations. Currently, the most popular POCT devices are based on methods of lateral flow immunochromatographic assay (ICA) which typically carry out the tests on chromatographic paper by capillary action. However, the batch differences in the porosity of papers will affect the precise flow of fluid and hence the accuracy and reproducibility of the results. On the contrary, microfluidic chips have more reproducible flow control over ICA due to the controllable material surface and microfluidic chip design. However, flow control still remains a major technical hurdle in microfluidics-based POCT devices [1]. Currently, flow control is mainly achieved by active pumping methods with bulky and complicated external parts. Hence, the development of power-free microfluidic chips to introduce samples and reagents is in high demand. In the past, various power-free microfluidic chips have been reported [1,2,3,4]. Nevertheless, the use of a microfluidic chip alone still involves multi-step operations to introduce a sample, mix the sample and several reagents for reactions, and detect the signals. This either requires a complicated microfluidic chip design or necessitates some user-unfriendly steps to be performed outside the microfluidic chip. In case of weak signals, sophisticated detection platforms are often used to boost the sensitivity, which weakens the purpose of POCT. Therefore, integration of a power-free microfluidic chip with a label-free biosensor is a promising combination to pave the way for POCT devices.

Since high-sensitivity and rapid biosensing technologies are highly desirable for the development of POCT devices, nanoplasmonic biosensors have become good choices. Most nanoplasmonic biosensors employ gold nanoparticles (AuNP) as the sensing material. The interaction between an incident light of specific frequency and AuNPs produces the phenomenon particle plasmon resonance (PPR), also known as localized surface plasmon resonance (LSPR), which is characterized by the extinction spectrum of the nanoparticle. The resonance frequency and the peak absorbance of the PPR band change considerably even when little variation in the local refractive index (RI) occurs on the nanoparticle surface [5,6]. This implies that when a bioreceptor is immobilized on the AuNP surface, the interaction between an analyte and the bioreceptor will increase the local RI of the medium surrounding the AuNP. Such a characteristic allows label-free and real-time monitoring of the interaction between the analyte and the bioreceptor, giving nanoplasmonic sensors the label-free and real-time detection capabilities. To further enhance the sensitivity, a fiber optic particle plasmon resonance (FOPPR) biosensor has been developed [7]. The principle of the FOPPR biosensor relies on the absorption of evanescent waves by AuNPs on the unclad segment of an optical fiber with an added advantage of signal enhancement by multiple total internal reflections (TIRs) of light along an optical fiber via the increase in optical path length and field magnification at the fiber core surface [7,8,9]. Therefore, after multiple TIRs, the light transmitted through the fiber is attenuated by interaction with the AuNPs. The FOPPR biosensor has been applied to quantitative analysis of numerous different species in many different fields such as environmental analysis (e.g., heavy metal ions [7,10,11]), medical diagnostics (e.g., antinuclear antibodies [12], cytokines [13,14], sepsis biomarkers [15], mRNA [16], single nucleotide polymorphism [17]), food safety analysis (e.g., staphylococcus enterotoxin B [8], organophosphorous pesticides [18], antibiotics [19]), forensic science (e.g., illicit drug [20]), and agricultural analysis (e.g., orchid viruses [21]). These experimental demonstrations indicate that the FOPPR biosensor has the benefits of wide linear dynamic range, good reproducibility, and low detection limit in the analysis of real samples.

In early December 2019, severe respiratory syndrome coronavirus 2 (SARS-CoV-2) was identified in Wuhan City, Hubei Province, China [22]. Thereafter, the number of 2019 coronavirus disease (COVID-19) infection cases went well above 480 million with over 6 million deaths and the numbers are still increasing day by day. The SARS-CoV-2 contains four main structural proteins, including nucleocapsid (N), spike (S), envelope (E), and membrane (M) proteins [23]. According to the amount of these proteins, nucleocapsid protein (N-protein) is the most abundant. It is also a relatively conserved protein in coronaviruses [24,25]. Hence, the SARS-CoV-2 antigen detection kits reported thus far primarily detect the N-protein [25]. On the contrary, the S-protein has evolved in recent months through mutations [26]. These unique features of the N-protein have made it a promising candidate for the development of COVID-19 diagnosis.

Rapid and reliable tests are crucial to suppress the threats of further spreading this already pandemic disease. Therefore, developing simple and rapid testing devices to deliver reliable and accurate virus detection results is urgently needed because of tremendous global demands to combat the COVID-19 pandemic. Currently, virus RNA tests using the real-time quantitative polymerase chain reaction (qRT-PCR) method have been a standard technology to accurately quantify the amount of SARS-CoV-2 in collected samples [27,28]. However, performing qPCR protocols requires tedious procedures lasting hours using sophisticated equipment in central laboratories by skilled personnel. These limitations are severe hurdles in the applicability of PCR-based methods to become rapid POCT methods. Although other recent inventions in experimental stages can carry out nucleic acid amplification tests in a couple of hours using simpler portable equipment, these devices often compromise their analytical sensitivity by skipping heating and cooling cycles in PCR procedures. Furthermore, according to the experience of handling severe acute respiratory syndrome (SARS), the PCR technology is probably not sensitive enough to detect the virus in secretions or serum until 3 days after the onset of symptoms [29].

Serological assay is another established diagnostic method for COVID-19. This method measures the neutralizing antibody level to the virus in the blood serum [30]. However, the major limitation of this method is that it only works for suspected patients who must have an immune response to SARS-CoV-2. Usually, the process of generating antibodies by the infected host only starts a few days after infection. Therefore, this approach does not confirm the presence of active virus but is particularly attractive for surveillance and epidemiological assessment at a population level.

SARS-CoV-2 antigens have been shown to be detectable in serum, urine, and mucous membranes of patients with early COVID-19 infection [31]. Thus, immunoassays for antigens belong to another class of diagnostic method for COVID-19. The most popular technique to detect viral protein antigens is enzyme-linked immunosorbent assay (ELISA) methods. Although ELISA has been proven to be a very robust and sensitive technique, performing ELISA requires tedious steps involving multi-washing and reagent conjugation. Thus, ELISA requires skilled personnel to minimize false positive and false negative results. In addition, ELISA is difficult to perform on site. Thus, the POCT applications by ELISA for early detection of SARS-CoV-2 are not feasible. Recently, an ELISA-based biosensing system integrating immunomagnetic bead technology and fiber-optic array for high-throughput detection of the SARS-CoV-2 N-protein was developed [32]. The method shortens the procedures to within 45 min, but it still requires the tedious steps typically used in ELISA. Moreover, various highly sensitive platforms using sophisticated instrumentation for detection of the SARS-CoV-2 N-protein were also developed in recent times [33,34], but they can hardly become a viable method accessible to POCT applications.

Currently, rapid antigen diagnostic tests (RADTs) based on lateral flow ICA can be quickly and easily used on site, but they suffer from low analytical sensitivity and insufficient reproducibility. Therefore, the development of ICA readers is still required and some special readers have been reported [35,36,37]. Electrochemical biosensors for detection of the SARS-CoV-2 N-protein have also been reported [38,39,40,41,42,43,44]. However, the requirement of a reference electrode in electrochemical biosensors may become a weak point as the liquid junction error often exists in practical situations [45]. In addition, the majority of low-cost electrochemical biosensors have limited accuracy and repeatability [42]. Optical sensors do not require a so-called reference optrode and also have the advantages of immunity to electromagnetic interference and higher multiplexing capability [46]. Today, only a few antigen tests have been EUA approved as POCT devices, in contrast to the EUA approval of more than 100 serology tests [47]. Therefore, it is imperative to develop new concepts of RADTs to include features such as rapid detection, high analytical sensitivity, good specificity to SARS-CoV-2, convenience, and easy management of hazardous waste. These RADTs are faster than PCR techniques and usually provide results in a few minutes. However, because no amplification of the target is involved, antigen tests are inherently less sensitive than PCR techniques. Hence, development of more sensitive techniques for antigen tests is highly desirable.

Antigen tests typically employ specific monoclonal antibodies to detect the SARS-CoV-2 structural proteins. However, low production yields of these recognition antibodies are difficult issues to be overcome, especially when producing these antibodies in large scale is required for commercial purposes. In addition, most antibody probes cannot remain stable for long period of time under either room temperature or frozen conditions. This durability problem often results in product storage complications. Therefore, an emerging class of recognition molecules called aptamers becomes an alternative.

Aptamers, also termed “chemical antibodies”, are a class of nucleic acid sequences that have high selectivity and affinity toward their targets [48]. In vitro selections of synthesized aptamers can be achieved using the standard systematic evolution of ligands by exponential enrichment (SELEX) procedures. Aptamers are synthetic nucleic acids and can be produced in large scale by chemical synthesis with extreme accuracy and reproducibility. Nucleic acid polymers are robust in nature and can be stored at room temperature for years. Moreover, by simple chemical modifications, the properties of an aptamer such as stability, affinity, and specificity can be enhanced. As such, these aptamer-based biosensors, often called aptasensors, have a high potential to be developed as POCT devices as they are more time-effective, cost-effective without batch-to-batch difference, and conveniently operated without complicated storage issues.

This research aims to combine the attractive features of a nanoplasmonic biosensor with high sensitivity plus label-free and real-time detection capabilities, a microfluidic chip with power-free auto-flowing function and self-contained unit for encapsulation of the infectious sample, and an aptamer with high specificity for the potential application in the detection of SARS-CoV-2. The N-protein was used here as a model target. As four single-stranded DNA (ssDNA) aptamers had been identified as candidates for binding to the N-protein of SARS-CoV-2 [49], they were investigated in this study for their binding kinetic constants and equilibrium dissociation constants with the N-protein. Then, the ssDNA aptamer with the highest affinity was selected as the recognition molecule for the development of a rapid biosensor for COVID-19 as an alternative to immunosensors.

In this research, the commercialized light-sensing Biomarker Analyzer INB-D200 and power-free sensor chips developed by Instant NanoBiosensors Co., Ltd. were used. The sensor chip is a microfluidic chip with auto-flowing function which allows the loading of fluid samples without connecting to an external power supply and enables the loading and detection of multiple fluid samples. To the best of our knowledge, this is the first report of a power-free microfluidic chip based on the balance between the gravitational potential and the capillary force to control fluid loading. Moreover, the sensor chip has a self-contained design to minimize the risk of virus leaking to the environment and is thus particularly suitable for POCT of infectious viruses. These characteristics together with the features of ease-of-use and portability will fit the trend of telemedicine where remote patient monitoring by providing rapid and convenient diagnostic results is becoming more and more important.

## 2. Materials and Methods

### 2.1. Reagents

The following reagent-grade chemicals were purchased from Sigma–Aldrich: Hydrogen tetrachloroaurate trihydrate (HAuCl_4_·3H_2_O), 11-mercaptoundecanoic acid (MUA; ≥95%), 6-mercapto-1-hexanol (MCH; ≥97%), 1-ethyl-3-(3-dimethylaminopropyl)-carbodiimine hydrochloride (EDC), N-hydroxy-succinimide (NHS), and ethanolamine. All aqueous solutions were prepared in ultrapure water from a Millipore Milli-Q water purification system with a specific resistance of 18.2 MΩ. Phosphate buffer saline (PBS) solution was used as a buffer to prepare standards. Nucleocapsid protein (N protein) (47.08 kDa) was obtained from Peptidecham Biotech Co., Ltd. (Kaohsiung, Taiwan). The four 58-nucleotide sequence SARS-CoV-2-NP aptamers for N protein as shown in Table 1, NP-A48, NP-A58, NP-A61, and NP-A15, were obtained from Anhui Aptamy Biotechnology Co., Ltd. (Hefei, China). They were identified and screened by SELEX. The real samples from nasopharyngeal swaps were obtained from Boca Biolistics (Pompano Beach, FL, USA) with the quantitative real-time reverse transcription polymerase chain reaction assay (qRT-PCR) results validated.

### 2.2. Biosensing System

**Figure 1:**
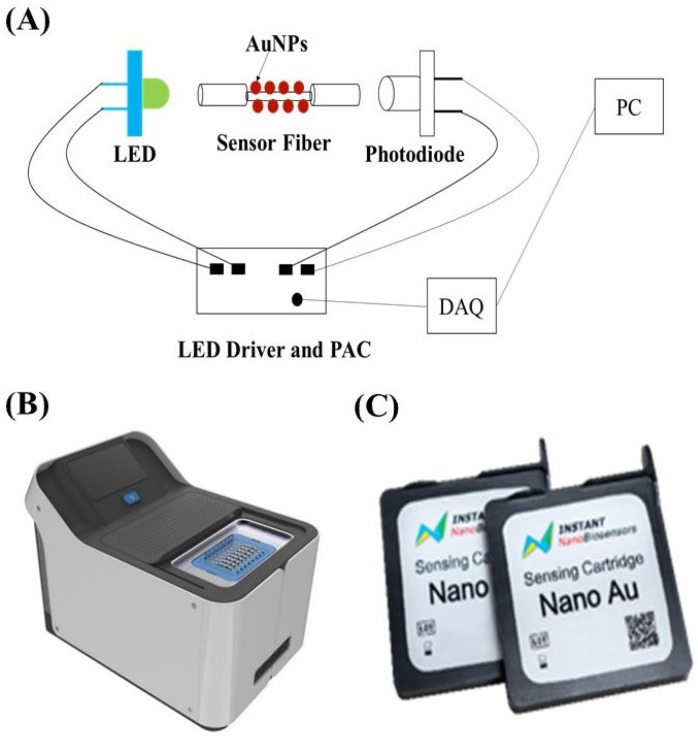
(**A**) Schematic representation of the modules used in the light-sensing Biomarker Analyzer INB-D200 biosensing system; (**B**) photograph of the commercialized product light-sensing Biomarker Analyzer (INB-D200) developed by Instant NanoBiosensors Co., Ltd.; (**C**) photograph of the auto-flowing sensor chips developed by Instant NanoBiosensors Co., Ltd. for sample analysis.

The commercialized product light-sensing Biomarker Analyzer (INB-D200) was developed by Instant NanoBiosensors Co., Ltd. (Taipei, Taiwan). A schematic of INB-D200 is shown in Figure 1A, which comprises a light source module consisting of a light-emitting diode (LED, specific peak wavelength = 522 nm, spectral bandwidth (FWHM) = 40 nm) connected to a LED driver, a chip loading module which can accommodate two sensor chips, a detection module consisting of two photodiodes (PD, spectral response range from 320 nm to 1100 nm) individually connected to a photoreceiver amplification circuit (PAC), a data acquisition (DAQ) module, and an external connected personal computer (PC). A software loaded in the PC is used to control the operations in INB-D200, receive data from INB-D200, and analyze the collected data.

A photograph of INB-D200 is shown in Figure 1B and a photograph of the sensor chips (NanoAu-MM) is shown in Figure 1C. For this biosensing system, the relative standard deviation (RSD) of the background noise is 3 × 10^−5^ on average of 300 s. Before any biosensing experiments, the system stability is checked first using this background noise RSD value. To ensure the signal has reached the steady state during the reaction in a bio-interaction analysis, the RSD value of the signal on average of 300 s is monitored by the system. When the RSD value is lower than 7 × 10^−5^, a green indicator light will be on to inform the user.

### 2.3. Sensor Chip and Test Protocol

The light-sensing Biomarker Analyzer sensor chip (NanoAu-MM) was developed and manufactured by Instant NanoBiosensors Co., Ltd. The sensor chip contained a multi-mode plastic clad silica optical fiber with core and buffer coating diameters of 400 and 730 μm, respectively, and with the removal of 20 mm cladding and buffer in the central part of the optical fiber. It is a general-purpose sensor chip with carboxyl groups on the AuNP surface to allow conjugation of a bioreceptor of interest. For most biomolecular detection applications with NanoAu-MM chip, the surface modification and calibration curve establishment procedures followed the recommended protocol. First, the sensing surface in the detection section should be sufficiently wetted by pipetting 80 μL ultrapure water before use. Second, to conjugate a ssDNA aptamer on the AuNP surface, the carboxyl groups on the AuNP surface were activated by an EDC/NHS solution (pH 7.0) for 20 min. Third, the ssDNA aptamer with terminal amine group was allowed to react with one activated carboxyl group on the AuNP surface via amine coupling by pipetting a 1 μM aptamer solution into the chip. Fourth, a 1 M ethanolamine solution (pH 8.5) was pipetted into the chip to deactivate excessive reactive groups on the AuNP surface for 10 min. For each loading step, the introduced fluid (80 μL) was kept in the microchannel of the detection section in the static mode until the next loading step. The flow time of the solution from the injection section to fill up the detection section was about 11 s. Before analysis, a blank buffer solution (1× PBS Buffer, pH 7.4) was used to establish a baseline for subsequent detection of the analyte in samples. For construction of calibration curves, five to six 80 μL standard samples with known concentration in 1× PBS Buffer at pH 7.4 were pipetted into the sensor chip sequentially from low to high concentration. Each sample typically needs 10 to 20 min to acquire the steady state sensor signal.

### 2.4. Method to Calculate Binding Kinetic Constants

Using the bioreceptor–analyte interaction model for FOPPR biosensor as previously described [50], a ssDNA aptamer was immobilized on the AuNP surface as a bioreceptor and the free N-protein was the analyte. Then, an N-protein solution was loaded into the chip to contact with the ssDNA aptamer on the AuNP surface. The reaction dynamics between the aptamer and the protein can be monitored by the temporal change of signal intensity. This transient intensity evolution can be plotted as a sensorgram. In the aptamer–protein interaction model, the formation of aptamer–protein complex on the AuNP surface can be described as

(1) $$\begin{array}{c} \;\;\;k_{a}\\ \mathrm{Aptamer}\;+\;\mathrm{Protein}\;⇌\;\;\;\mathrm{Aptamer}-\mathrm{Protein}\\ \;\;\;k_{d} \end{array}$$

In Equation (1), *k_a_* and *k_d_* are the association rate constant and the dissociation rate constant, respectively, governing the formation of the product, aptamer–protein complex. Since the immobilized ssDNA aptamer binds with the protein to form an immobilized aptamer–protein complex on the AuNP surface, the model in Equation (1) is similar to the Langmuir isotherm when the aptamer-protein complex is assumed to pile as a monolayer.

## 3. Results and Discussion

### 3.1. Principle of Label-Free Detection by FOPPR Biosensor

**Figure 2:**
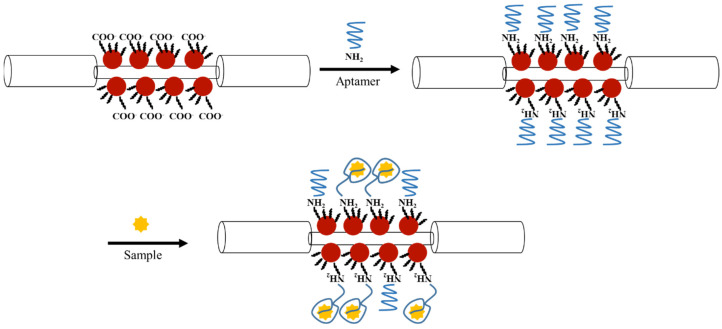
Schematic illustration of the reactions in bioconjugation of aptamer and the binding between the immobilized aptamer on AuNPs and the N-protein in sample.

The principle of FOPPR sensing technology is based on the penetration of evanescent wave in the absorbing medium outside the fiber core and the overall sensitivity depends on the length of sensing region on the fiber core surface [8], the surface coverage of AuNPs in the sensing region [9], and the affinity between the analyte and the immobilized receptor [51]. Figure 2 shows a schematic illustration of the label-free detection of the N-protein by an aptamer-functionalized sensor fiber using FOPPR biosensor. When light of appropriate frequency propagates along the optical fiber, the evanescent field excites the AuNPs, leading to a nanoplasmonic effect while the excitation of the guided modes in TIR immensely enhances light/matter interaction and the multiple TIRs increase the optical path length. Thus, the light is attenuated by interaction with AuNPs and the attenuation is further enhanced by the multiple TIRs, resulting in significant increase in sensing sensitivity. Since molecular binding at the AuNP surface will result in an increase in local refractive index (RI) at the AuNP surface and hence an increase in the extinction cross-section of the AuNP [13], the transmitted light intensity through the optical fiber will decrease, enabling real-time observation of the molecular binding event. Quantitative analysis in this study is implemented by comparing the transmitted light intensity of a sensor fiber immersed in a sample solution (I_s_) containing a fixed or unknown concentration of target protein to that immersed in a blank solution without the target protein (I_0_) and the sensor response is defined as (I_0_ − I_s_)/I_0_ = ΔI/I_0_. 

### 3.2. Power-Free Microfluidic Chip

**Figure 3:**
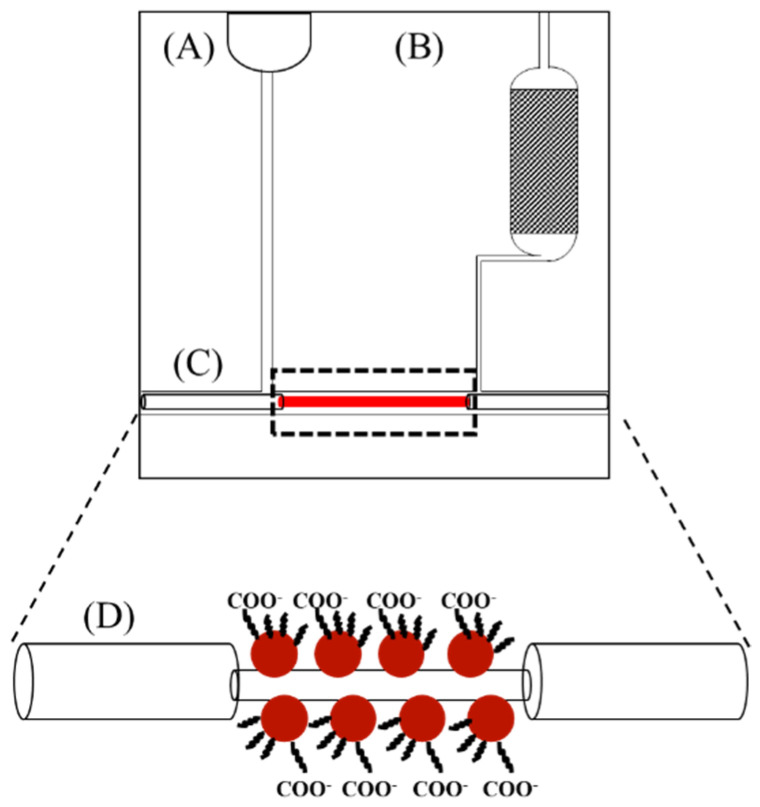
A schematic of the microfluidic sensor chip (NanoAu-MM): (**A**) Fluid injection section; (**B**) fluid storage section, where the porous material is located in the shaded area; (**C**) detection section; and (**D**) exploded view of the sensor fiber in the detection section (**C**).

The microfluidic chip comprises a fluid injection section, a detection section which accommodates an optical fiber with AuNPs coated in the unclad region, and a fluid storage section which contains a porous absorbent material to absorb the drainage liquid from the detection section, as showed in Figure 3. The porous absorbent material, for example, can be made of polysulfone, cellulose ester, polyvinyl alcohol, or polyacrylate. This power-free auto-flowing sensor chip can load fluid samples without a driving device since fluid flow is driven by gravity. When the first fluid sample is loaded into the fluid injection section, it is driven by gravity to pass through the detection section and accumulates to form a droplet at the fluid inlet of the fluid storage section, such that the gravitational potential due to the height difference between the fluid outlet of the fluid injection section and the fluid inlet of the fluid storage section is equal to the capillary force opposite to the direction of gravity of a portion of the fluid sample, and thus a state of fluid pressure equilibrium is established. In the fluid storage section, a spacing section is defined to prevent the contact between the droplet at the fluid inlet of the fluid storage section and the porous absorbent material so that the droplet is only allowed to accumulate up to a certain size. When the droplet at the fluid inlet of the fluid storage section gradually increases in size until it is in contact with the porous absorbent material, the excess amount of fluid will be absorbed by the porous absorbent material, thereby maintaining the droplet at the pre-defined size and its contact with the porous absorbent material is again cut off.

When a second fluid sample is loaded into the fluid injection section, it drives the first fluid to leave the detection section and then it passes through the detection section until the gravitational potential and the capillary force are again balanced. It should be noted that the amount of porous absorbent material disposed in the vicinity of the fluid inlet of the fluid storage section allows the loading of at least six fluid samples. Therefore, a standard calibration curve can be constructed by using just one microfluidic chip. Thus, this microfluidic chip allows self-pumping by gravity without connecting to an external power supply and enables the loading and detection of multiple fluid samples. Furthermore, this microfluidic chip has a self-contained design which is particularly suitable for POCT of infectious samples. After the sample from a patient is loaded into the chip to take measurement, it will be absorbed in the chip. To avoid the risk of the virus leaking to the environment, the sample contained in the chip can be disinfected by further loading a disinfectant agent into the chip to make sure the used chip is risk-free.

### 3.3. Reproducibility in Bioconjugation of ssDNA Aptamer

**Figure 4:**
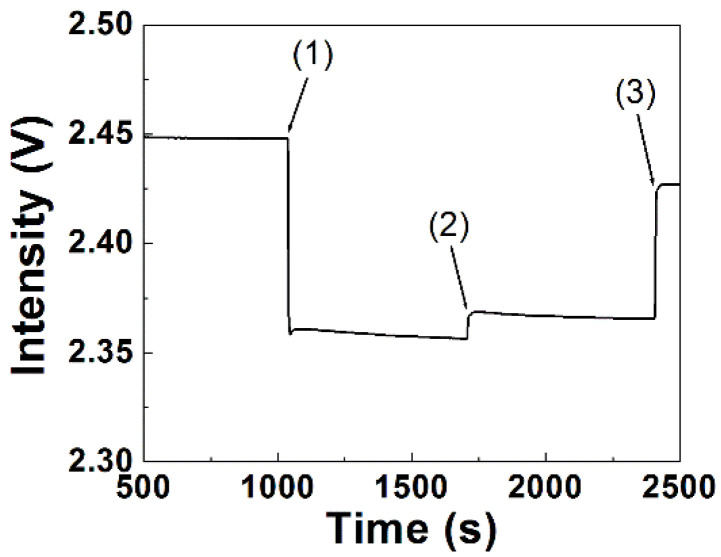
Typical real-time sensorgram during the surface activation process of aptamer NP-A48. Steps 1, 2, and 3 indicate loading of first EDC/NHS solution, second EDC/NHS solution, and ultrapure water, respectively.

As the reproducibility in bioconjugation of a recognition element is one of the major factors affecting the reproducibility of a biosensor, we examine the reproducibility of our bioconjugation process in this power-free microfluidic chip by analyzing the FOPPR sensor responses. Before conjugation of a ssDNA aptamer on AuNPs, an EDC/NHS solution was used to activate the carboxyl groups on the AuNP surface. As the FOPPR sensor response is very sensitive to the changes of both the local RI near the AuNP surface and the bulk RI, the sensorgram during the surface activation process serves as a monitoring tool. As shown in Figure 4, when an EDC/NHS solution was loaded into a sensor chip, a step decrease in signal intensity at the beginning was observed due to a big change in bulk RI from 1.333 to 1.335. Then, a gradual decrease in signal intensity can be observed due to a local RI change near the AuNP surface caused by the activation reaction. To ensure complete surface activation, a second EDC/NHS solution was loaded again. Due to the exothermal nature of the EDC/NHS reaction, the loading of the cooler second EDC/NHS solution leads to a small increase in signal intensity since the FOPPR response is weakly dependent on the temperature of the bulk solution [15]. After the activation process, ultrapure water was loaded into the chip to wash away excess EDC and NHS for subsequent biosensing experiments. The signal intensity at this step as compared to that before surface activation then serves as an indicator of the successfulness of the surface activation process. For example, a sensor response of 0.0086 for the surface activation process was observed in Figure 4.

**Figure 5:**
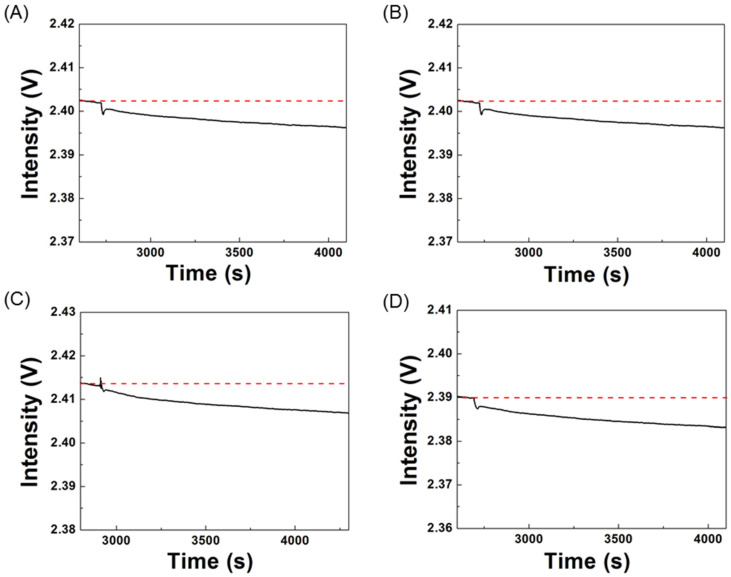
Typical real-time sensorgrams during the bioconjugation process of ssDNA aptamers: (**A**) NP-A48, (**B**) NP-A58, (**C**) NP-A61, and (**D**) NP-A15. Aptamer concentration = 1 μM. The black solid lines indicate the real-time signals and the red dot lines are tangent lines drawn from the baselines.

To compare the number of immobilized biomolecules from chip to chip, the signal intensities before and after the bioconjugation process can be utilized to monitor the amount of the four ssDNA aptamers being immobilized, since in principle the sensor response is directly related to the change of effective local RI due to specific adsorption [52]. As shown in Figure 5, the signal intensities during the bioconjugation process of the four ssDNA aptamers gradually decrease due to the local RI increase near the AuNP surface caused by the covalent attachment of the ssDNA aptamers to the AuNP surface. Table 2 summarizes the average, standard deviation (SD), and coefficient of variation (CV) of the sensor responses in bioconjugation of the four ssDNA aptamers. The results shown in Table 2 indicate that the reproducibility in bioconjugation of the ssDNA aptamers is very good with CV values fall in the range of 2.6% to 7.7%. 

### 3.4. Estimation of Binding Affinity and Kinetic Constants

**Figure 6:**
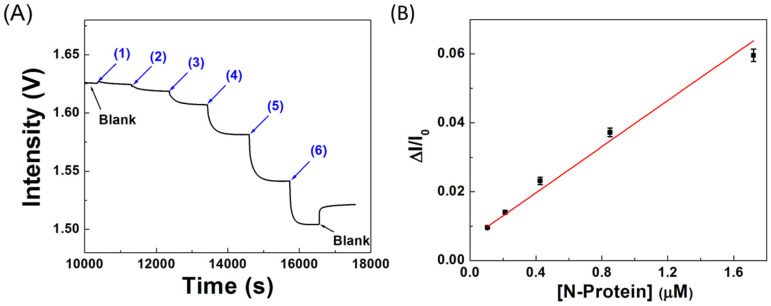
(**A**) Real-time sensorgram of sequentially pipetting standard N-protein solutions with different concentrations of (1) 53 nM, (2) 106 nM, (3) 212 nM, (4) 424 nM, (5) 848 nM, and (6) 1.7 µM to a NP-A48 aptamer-functionalized sensor chip; (**B**) the corresponding standard calibration curve (*n* = 2).

Measurement of the rate and affinity of biomolecular interactions provides quantitative information which is beneficial to the development of diagnostic techniques. As described in Equation (1), each N-protein molecule binds with one immobilized ssDNA aptamer at the AuNP surface, where the association and dissociation rate constants of forming the aptamer–protein complex are *k_a_* and *k_d_*, respectively. At the beginning of the binding reaction, the N-protein molecules are not yet depleted even in the regime near the binding surface, so that the N-protein concentration remains virtually unchanged and is almost the same as the bulk concentration. When this condition is still valid, the binding kinetics are controlled by the association and dissociation rate constants while the mass transport effect of the N-protein molecules to the sensor surface is negligible. In this circumstance, the rate of product complex formation will follow the pseudo-first-order rate equation as previously described [50]. Figure 6A shows a typical real-time sensorgram of the binding reaction between NP-A48 aptamer and free N-proteins.

According to Equation (1), the concentration of the product, aptamer–protein complex, can be related to the light intensity I_t_, where I_t_ is the light intensity at time t after loading a target protein solution into a ssDNA aptamer-functionalized senor chip. As shown in Figure 6A, by sequentially loading a series of solutions from low to high concentration with specific concentration C_i_, the plot of the logarithm form of ln[(I_t_ − I_eq_)/(I_0_ − I_eq_)] versus time t should be linear, where I_0_ represents the initial transmitted light intensity of a sensor fiber in a blank solution where all binding sites are available, and I_eq_ represents the steady state light intensity when the reaction between the immobilized ssDNA aptamer and the target protein reaches equilibrium. From the plot of ln[(I_t_ − I_eq_)/(I_0_ − I_eq_)] versus time t at a specific concentration C_i_, the slope of the linear regression line should be equal to S = −(*k_a_* · C + *k_d_*). Therefore, by loading several sample solutions with known original concentrations C_1_, C_2_, …, and C_i_ consecutively into the sensor chip, the resulting slopes can be calculated individually as S(C_1_), S(C_2_), …, and S(C_i_). By using at least three different concentrations of the N-protein, the binding kinetic constants and hence the equilibrium dissociation constant (K_D_) can be calculated for the four different ssDNA aptamers. 

**Figure 7:**
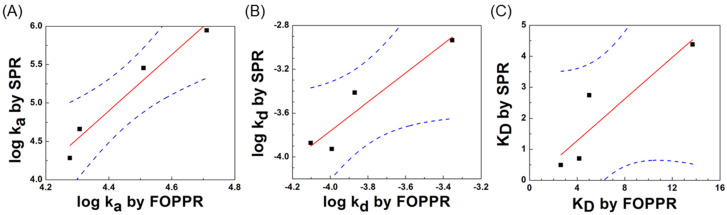
The linear correlations for (**A**) association rate constant, (**B**) dissociation rate constant, and (**C**) equilibrium dissociation constant, between the results obtained by FOPPR biosensor and SPR biosensor for the binding of each of the four ssDNA aptamers with the N-protein. Red solid lines are regression lines and black dashed lines are the 95% confidence intervals for prediction with the linear regression model.

Using the slopes of the linear regression lines obtained for NP-A48 aptamer with the N-protein, the association rate constant *k_a_*, and dissociation rate constant *k_d_* were calculated to be 5.14 × 10^4^ M^−1^s^−1^ and 1.35 × 10^−4^ s^−1^, respectively. Similarly, the association and dissociation rate constants for NP-A58, NP-A61, and NP-A15 with the N-protein were calculated and listed in Table 2. Furthermore, the affinity between each binding pair can be revealed by K_D_ which can be calculated from the ratio of *k_d_* to *k_a_*. Table 3 lists the calculated results of the four sets of kinetic rate constants *k_a_* and *k_d_* and the equilibrium dissociation constant K_D_ using the sensorgrams obtained from the binding between each of the ssDNA aptamers and the N-protein. Such results are also compared with those obtained by SPR sensor previously reported [49]. As shown in Figure 7, the linear correlations for association rate constant, dissociation rate constant, and equilibrium dissociation constant between the results obtained by FOPPR biosensor and SPR biosensor are good, with correlation coefficients of 0.98, 0.95, and 0.91, respectively, indicating that the results obtained by the two technologies are highly correlated. Furthermore, statistical analysis of the results from the two methods yields *p*-value < 0.01 (*p*-value was 0.0056) at the 95% significance level, further supporting that the results obtained by both methods agree with each other.

In the SPR sensor chip, the dense polymer coating layer has been suggested to encounter a few problems, such as retention effect and rebinding [53,54]. Because of this dense layer on the SPR sensor chips, when the N-protein molecules diffuse into this layer to interact with the immobilized aptamer, the diffusion is hindered by the coating layer. When the N-protein molecules dissociate from the surface and diffuse out of the layer, the diffusion is again hindered. Since these N-protein molecules are easier to diffuse into rather than diffuse out of this dense layer, they are stacked inside the coating layer. Therefore, the N-protein molecules stacking inside the layer cause the rebinding effect, thus altering the binding kinetics and resulting in the overestimated association rate constant *k_a_*. On the other hand, FOPPR biosensor does not have a dense polymer coating layer on the fiber core surface. Therefore, the protein molecules will not stack on the surface, and hence, the effect of rebinding of dissociated N-protein molecules back onto the AuNP surface is minor. As can be seen in Table 3, *k_d_* values estimated by FOPPR biosensor and SPR biosensor are close, while the *k_a_* values estimated by SPR biosensor are larger than that by FOPPR biosensor.

### 3.5. Specificity Tests

**Figure 8:**
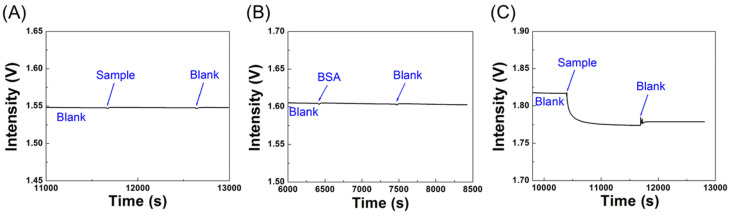
Real-time sensorgrams of NP-A48-functionalized sensor chips in response to (**A**) a solution of S-protein (1 μg/mL), (**B**) a solution of BSA (1 μg/mL), and (**C**) a solution of the N-protein (1 μg/mL).

The specificity of the biosensor was determined by employing an NP-A48-functionalized sensor chip in response to solutions of the N-protein (1 μg/mL), S-protein (1 μg/mL), and bovine serum albumin (BSA). The detection section in the sensor chip was firstly filled with a blank PBS buffer. Then, a solution of the N-protein, BSA, or S-protein was introduced into the sensor chip. As shown in Figure 8, I_0_ was used to validate the system stability first. When a sample of the S-protein was loaded into a sensor chip, I_S_ as shown in Figure 8A was indistinguishable from the baseline, suggesting NP-A48 aptamer does not bind with the S-protein. Similarly, when a solution of BSA was loaded into a sensor chip, I_S_ was indistinguishable from the baseline as shown in Figure 8B. On the other hand, when a sample of the N-protein was loaded into a sensor chip, I_S_ as shown in Figure 8C decreased with time and followed a molecular binding kinetic curve [50], suggesting that NP-A48 aptamer does bind with the N-protein specifically. The sensor response time, when defined as the time to reach 95% of the steady state signal intensity, is about 900 s. From these results, the specificity of NP-A48 aptamer to the N-protein is confirmed.

### 3.6. Sensitivity of the Biosensor

As shown in Table 3, NP-A48 aptamer has the highest affinity and favorable kinetic rate constants among the four aptamer candidates. Therefore, it was selected as the recognition molecule to develop a rapid biosensor for the N-protein of SARS-CoV-2. The sensitivity of the biosensor was determined by employing an NT-A48 aptamer-functionalized sensor chip in response to solutions of the N-protein of increasing concentration to construct a standard calibration curve. The sensor chip was firstly filled with PBS buffer as a blank. Next, standard samples of the N-protein with increasing concentration from 53 nM to 848 nM were sequentially introduced into the sensor chip. For each standard sample, the solution was kept in the microchannel of the sensor chip for 15 min in the static mode. The real-time sensorgram for sequential loading of the N-protein solutions with increasing concentration is shown in Figure 6A, where the steady state signal intensity (I_s_) for each sample decreases when the N-protein concentration increases. The calibration curve as shown in Figure 6B was constructed by using the I_s_ values corresponding to each concentration of the N-protein. The linear regression equation of the calibration plot is *y* = 0.00631 + 0.03343x with a correlation coefficient of 0.993. The method is highly reproducible with CV values less than 6% over the whole concentration range. From the calibration curve, the limit of detection (LOD, with signal-to-noise ratio = 3:1) for the N-protein was calculated to be 2.8 nM (1.3 × 10^−7^ g/mL). Based on a method correlation study from the literature [16], it was shown that the Ct value of 39.7 (≈95 copies/mL) by qRT-PCR is equivalent to the N-protein concentration of 0.71 ng/mL. By this conversion factor, our LOD is equivalent to about 1.5 × 10^4^ copies/mL, which meets the acceptable LOD of 10^6^ copies/mL set by the WHO target product profile [55]. 

Although our biosensor is inferior to some of the sophisticated biosensing platforms [15,16], it is label-free and the analysis can be completed within 15 min. Together with the advantages offered by aptamers and the potential of being developed as a portable device, our biosensor is particularly suitable for POCT applications. Recently, several label-free aptasensors for COVID-19 antigen tests with similar or slightly better LOD were reported [56,57,58], but they target the spike protein (S-protein) rather than the N-protein. In comparison to a study using either sandwiched aptamers or aptamer-antibody hybrid in ELISA or CIA for the N-protein [49], our biosensor using a single aptamer exhibits similar LOD. By using our recently developed fiber optic nanogold-linked sorbent assay (FONLISA) which has been demonstrated to have LOD at least three orders lower than the present label-free direct assay [8], we expect a significant improvement in LOD to the pM level. Given the advantages of FOPPR biosensor including ease of operation, high analytical sensitivity, wide linear dynamic range, low-cost instrumentation and sensor chip, power-free and self-contained sensor chip design, and scalability in mass production, it could be an attainable alternative for POCT of infectious viruses.

### 3.7. Real Sample Tests

**Figure 9:**
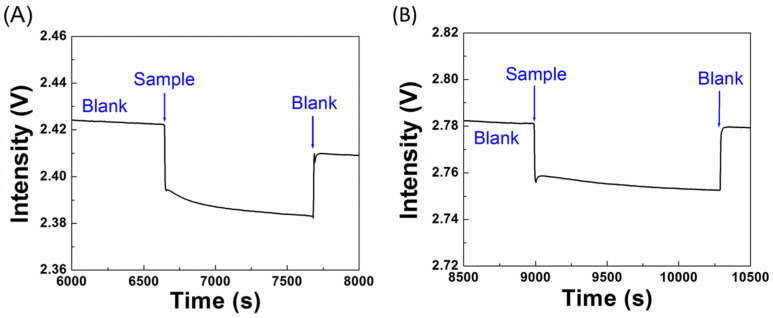
Real-time sensorgrams of a NP-A48-functionalized sensor chip in response to (**A**) a negative sample spiked with 5 μg/mL (0.11 μM) N-protein and (**B**) an unspiked negative sample.

To investigate the feasibility of applying the biosensor in real samples, we spiked a known concentration of the N-protein into negative samples of nasopharyngeal swabs to determine the recovery. The protocol to analyze real samples followed the Interim Guidelines for Collecting and Handling of Clinical Specimens for COVID-19 Testing from CDC. First, a solution containing 50% lysis buffer and 50% PBS for lysing virus particles was prepared. Before sample loading, a 50 μL real sample was added into 50 μL lysis buffer for 15 min incubation. Then, a blank solution of PBS buffer and DMEM blank medium with 50% lysis buffer at the same volume ratio was used to establish the baseline. When the RSD value of the signal (I_0_) in the baseline is lower than 0.01% within 300 s, a 100 μL sample can be introduced into the chip. A typical sensorgram is showed in Figure 9A, where a solution of the N-protein (5 μg/mL, 0.11 μM) was spiked into a negative sample. It can be seen that a step decrease in signal intensity at the beginning was observed due to a big change in bulk RI. Then, a gradual decrease in signal intensity following a molecular binding kinetic curve [50] was observed due to a local RI change near the AuNP surface as a result of the binding of the N-protein with the immobilized NT-A48 aptamer. After the reaction reached steady state, i.e., when the RSD value of the signal fell below 7 × 10^−5^, a 100 μL blank solution was loaded into the chip to remove any non-reacted sample and to compensate for the effect of bulk RI change on the FOPPR signal. Then this signal (I_s_) was used to calculate the sensor response, which is calculated to be 0.00547. On the other hand, for an unspiked negative sample, a typical sensorgram is showed in Figure 9B. It can be seen that after a step decrease in signal intensity at the beginning, the molecular binding kinetic curve was not observed afterwards. Further loading of a blank yielded a sensor response of 0.00063, which is significantly smaller than that of the spiked sample. Using the calibration curve as shown in Figure 6B, the recoveries of two spiked tests were calculated to be 103% and 90%. Therefore, the biosensor can effectively determine the N-protein in human nasopharyngeal swap samples, suggesting the potential of the biosensor as a clinical tool for diagnosis of COVID-19.

## 4. Conclusions

The development of a sensitive, selective, rapid, and portable biosensor for early detection of infectious viruses is crucial especially during pandemics. This paper investigated a sensitive, rapid, and robust FOPPR biosensor for the detection of the SARS-CoV-2 N-protein based on an ssDNA aptamer-based biorecognition element. The biosensor has desirable characteristics for POCT of infectious viruses, including features such as label-free, real-time, high sensitivity, wide linear dynamic range, ease of operation, portable, low-cost instrumentation and sensor chip, and power-free and self-contained sensor chip design. As a proof of concept, the biosensor exhibits short analysis time (15 min) and LOD of 2.8 nM to meet the acceptable LOD of 10^6^ copies/mL set by the WHO target product profile. Use of spiked negative samples of nasopharyngeal swaps with known concentrations of the N-protein has demonstrated the potential of the method for clinical samples. By using the FONLISA method in FOPPR biosensor in the future, a significant improvement of the LOD to the pM level is expected. Due to the versatile design of the biosensor, the biorecognition element could easily be replaced to fit a new application, say, to detect mutant variants of the S-protein of SARS-CoV-2 by employing an updated aptamer or antibody. We anticipate that this biosensor platform has huge potential to be developed as a new method and a POCT device for rapid diagnostics of SARS-CoV-2 to control its outbreak.

## Figures and Tables

**Table 1:** The sequence of the ssDNA aptamers for the SARS-CoV-2 N-protein [49].

Aptamer	Sequence (5′ → 3′)
NP-A48	GCTGGATGTCGCTTACGACAATATTCCTTAGGGGCACCGCTACATTGACACATCCAGC
NP-A58	GCTGGATGTCACCGGATTGTCGGACATCGGATTGTCTGAGTCATATGACACATCCAGC
NP-A61	GCTGGATGTTGACCTTTACAGATCGGATTCTGTGGGGCGTTAAACTGACACATCCAGC
NP-A15	GCTGGATGTTCATGCTGGCAAAATTCCTTAGGGGCACCGTTACTTTGACACATCCAGC

**Table 2:** The sensor responses in bioconjugation of four ssDNA aptamers (aptamer concentration = 1 μM, *n* = 3).

	NP-A48	NP-A58	NP-A61	NP-A15
Average	0.00517	0.00145	0.00251	0.00440
SD	1.38 × 10^−4^	7.79 × 10^−5^	6.42 × 10^−5^	3.37 × 10^−4^
CV	2.7%	5.4%	2.6%	7.7%

**Table 3:** Comparison of association rate constant (*k_a_*), dissociation rate constant (*k_d_*), equilibrium dissociation constant (K_D_) for the binding between each of the four different ssDNA aptamers and the N-protein measured by FOPPR biosensor and SPR biosensor [49].

Aptamer	*k_a_* (M^−1^s^−1^) [FOPPR]	*k_a_* (M^−1^s^−1^) [SPR]	*k_d_* (s^−1^) [FOPPR]	*k_d_* (s^−1^) [SPR]	K_D_ (nM) [FOPPR]	K_D_ (nM) [SPR]
NP-A48	5.14 × 10^4^	8.80 × 10^5^	1.35 × 10^−4^	3.85 × 10^−4^	2.63	0.49
NP-A58	1.89 × 10^4^	1.92 × 10^4^	7.89 × 10^−5^	1.34 × 10^−4^	4.17	0.70
NP-A61	2.03 × 10^4^	4.58 × 10^4^	1.02 × 10^−4^	1.18 × 10^−4^	5.02	2.74
NP-A15	3.24 × 10^4^	2.84 × 10^5^	4.44 × 10^−4^	1.16 × 10^−3^	13.70	4.38

## Data Availability

All relevant data are available in the manuscript.
